# A System of Medicine

**Published:** 1912-03

**Authors:** 


### Reviews of Books.

---

**A System of Medicine.** By Many Writers. Edited by Sir Clifford Allbutt, K.C.B., M.A., M.D., and Humphry Davy Rolleston, M.A., M.D. Vol. IX. **Diseases of the Skin. General Index.** Pp. xviii, 869. London: Macmillan and Co. Limited. 1911.

It is almost a pity that this volume on Dermatology should to some extent lose its identity by being part of a System of General Medicine. There is a risk lest readers should be misled into supposing that the subject will not be exhaustively dealt with as a branch of the larger field. But there must be no room for such an error. The collection of monographs on skin diseases contained in this the concluding volume of Allbutt and Rolleston's *System of Medicine* has no rival in the English language.

On first looking through the volume we thought of it chiefly as a valuable reference work for dermatologists; but a better acquaintance with it has convinced us that the articles are written in so readable a style, the various diseases are so easy of reference, and treatment so admirable, that every practitioner should possess and keep handy Allbutt and Rolleston's *On Diseases of the Skin.*

